# Assessing the Emergence of Resistance: The Absence of Biological Cost *In Vivo* May Compromise Fosfomycin Treatments for *P. aeruginosa* Infections

**DOI:** 10.1371/journal.pone.0010193

**Published:** 2010-04-15

**Authors:** Alexandro Rodríguez-Rojas, María D. Maciá, Alejandro Couce, Cristina Gómez, Alfredo Castañeda-García, Antonio Oliver, Jesús Blázquez

**Affiliations:** 1 Centro Nacional de Biotecnología (CNB), Consejo Superior de Investigaciones Científicas (CSIC), Madrid, Spain; 2 Servicio de Microbiología and Unidad de Investigación, Hospital Son Dureta, Instituto Universitario de Investigación en Ciencias de la Salud (IUNICS), Palma de Mallorca, Spain; 3 Servicio de Anatomía Patológica, Hospital Son Dureta, Palma de Mallorca, Spain; The Scripps Research Institute, United States of America

## Abstract

**Background:**

Fosfomycin is a cell wall inhibitor used efficiently to treat uncomplicated urinary tract and gastrointestinal infections. A very convenient feature of fosfomycin, among others, is that although the expected frequency of resistant mutants is high, the biological cost associated with mutation impedes an effective growth rate, and bacteria cannot offset the obstacles posed by host defenses or compete with sensitive bacteria. Due to the current scarcity of new antibiotics, fosfomycin has been proposed as an alternative treatment for other infections caused by a wide variety of bacteria, particularly *Pseudomonas aeruginosa*. However, whether fosfomycin resistance in *P. aeruginosa* provides a fitness cost still remains unknown.

**Principal Findings:**

We herein present experimental evidence to show that fosfomycin resistance cannot only emerge easily during treatment, but that it is also cost-free for *P. aeruginosa*. We also tested if, as has been reported for other species such as *Escherichia coli*, *Klebsiella pneumoniae* and *Proteus mirabilis*, fosfomycin resistant strains are somewhat compromised in their virulence. As concerns colonization, persistence, lung damage, and lethality, we found no differences between the fosfomycin resistant mutant and its sensitive parental strain. The probability of acquisition *in vitro* of resistance to the combination of fosfomycin with other antibiotics (tobramycin and imipenem) has also been studied. While the combination of fosfomycin with tobramycin makes improbable the emergence of resistance to both antibiotics when administered together, the combination of fosfomycin plus imipenem does not avoid the appearance of mutants resistant to both antibiotics.

**Conclusions:**

We have reached the conclusion that the use of fosfomycin for *P. aeruginosa* infections, even in combined therapy, might not be as promising as expected. This study should encourage the scientific community to assess the *in vivo* cost of resistance for specific antibiotic-bacterial species combinations, and therefore avoid reaching universal conclusions from single model organisms.

## Introduction

Amongst a number of old antibiotics being used once more as appealing alternatives for the treatment of different infections, fosfomycin (Fos) displays some features that place it as a very promising compound [1]. Although its use has traditionally been focused on urinary tract infection pathogens, fosfomycin is a broad-spectrum bactericidal antibiotic, that is active against both gram-positive and gram-negative bacteria [2]. It has shown almost no toxicity and allergenicity in humans [3]. It displays little cross-resistance with other antibiotics, probably because it is chemically unrelated to any other known antimicrobial agent [2], [3]. Also, several studies have pointed out the synergistic effect of fosfomycin when used in combination with other antimicrobials [4], [5], [6], [7]. Fosfomycin treatments have shown a relatively low proneness of resistant mutants to persist *in vivo*, leading to a good effectiveness in the therapy, at least against *Escherichia coli* [8]. However, recently the acquisition of fosfomycin resistance in a previously circulating CTX-M-15-producing *E. coli* O25b-ST131-phylogroup B2 strain has been reported. This apparently occurred when the use of fosfomycin was increased by 50% [9].

The emergence of antibiotic resistance in a bacterial population is shaped by several factors, of which the mutation rate to resistance and the fitness cost of the mutants have been considered critical [10]. If resistance implies a considerable cost for the bacteria, their growth rate would not be enough to offset the wash out dynamic imposed by the diverse body fluids, the killing mediated by the immune system, or it might allow the more fit susceptible bacteria to out-compete the resistant ones once the antibiotic is removed [10].

The trend of resistant bacteria to have a competitive disadvantage in a untreated environment is related to the precise mechanism of the resistance in question. Numerous types of resistance achieved by chromosomal mutations are caused by target alterations [11]. In these cases a consistent fitness cost may appear if the resistant target displays a somewhat suboptimal functionality, as has been shown, for example, with some mutations in the gene *rpsL* conferring streptomycin in bacteria such as *E. coli* or *Salmonella spp.* [12]. Another prominent way to attain resistance is determined by accessory elements, where resistance is afforded by enzymes or pumps that inactivate or remove the antimicrobial from the cell [13]. This type of resistance may also give rise to fitness cost, as the replication and activity of the elements themselves could involve some metabolic expenditures [11].

In the specific case of fosfomycin, resistance is mainly acquired by reducing the cell uptake of the drug [14], [15], [16]. This kind of resistance has been widely shown to compromise *E. coli* at a slower rate of growth when compared to that of the sensitive parental strains [8], [17], [18], probably because most mutations disturb carbon metabolism [8]. In addition, and very conveniently for humans, these mutants also displayed a reduced virulence represented by lower adhesion to epithelial cells [18], [19], [20]. The data for other species are scarce, but it has been reported that *Klebsiella pneumoniae* and *Proteus mirabilis*, common bacteria also causing uncomplicated urinary tract infections, follow the same trend as *E. coli* [18]. Surprisingly, a new resistance mechanism based on amino acid substitutions in MurA, the target protein of fosfomycin, has recently been reported. Unfortunately, the authors have not estimated the cost associated to such a modification [21].

Recently it has been reported that the use of fosfomycin be extended in order to treat pathogens like *Pseudomonas aeruginosa* [2], where the emergence of antibiotic resistance has led to a unpleasant scarcity of treatments [22]. *P. aeruginosa* is one of the leading nosocomial pathogens worldwide. The most severe infections produced by *P. aeruginosa* occur in Intensive Care Unit patients, those suffering from chronic respiratory diseases and immunocompromised individuals [23]. Considering the example of *E. coli*, it seems reasonable to expect that fosfomycin might serve as a genuine alternative to treat infections caused by multidrug resistant *P. aeruginosa*. This possibility has been substantiated by published data showing that fosfomycin, furthermore, could reveal a particular effectiveness when used in combined therapy with other well-known antipseudomonal agents [2], [4], [5], [6], [7].

In a previous work, we reported that *P. aeruginosa* has a high mutation frequency to fosfomycin resistance *in vitro* [24]. These observations *in vitro* could undermine the good expectations created about fosfomycin. However, sometimes the data generated *in vitro* do not agree with the results attained *in vivo* [10], [25]. Here, we undertook the assessment of this disturbing possibility, monitoring the emergence of fosfomycin resistant (Fos-R) mutants in an *in vivo* murine model. We then checked the fitness cost caused by the inactivation of *glpT* in one insertion (null) mutant, as well as its virulence relative to the sensitive parental strain. Our results indicate a deviation from the *E. coli* model, disproving any connection between fosfomycin resistance, fitness cost or loss of any trait conferring bacterial virulence in *P. aeruginosa*.

## Methods

### Ethics Statement

All animal experiments were approved by the animal ethics committee of the University of the Balearic Islands, Spain. All animals were handled and housed in strict accordance with guidelines from the University of the Balearic Islands.

### Bacteria and media

The *P. aeruginosa* PA14 and its derivative mutants *mutS::MAR2xT7* and *glpT::MAR2xT7* were kindly provided by Dr. N. T. Liberati [26]. All the *P. aeruginosa* strains were cultured in Luria-Bertani (LB) or Mueller-Hilton at 37°C, and gentamycin (10 µg/ml) was added when appropriate. For the biofilm assay, LB and FBA minimal media were used [27].

### *P. aeruginosa* lung infection mouse model

The murine model of *P. aeruginosa* lung infection was established by intranasal inoculation of bacteria [28], [29], [30]. Briefly, for the preparation of the inocula, bacteria (*P. aeruginosa* strains PA14, PA14*mutS::MAR2xT7*, PA14*glpT::MAR2xT7* or a 1∶1 mix of PA14/PA14*glpT::MAR2xT7*) were grown in LB to 0.5 OD 600 nm, centrifuged at 10,000 g for two minutes and diluted 10-fold in saline solution (1 and 5-fold dilutions in saline solution were also tested during the standardization of the model). Finally, serial 1/10 dilutions of bacterial suspensions were plated in MHA to estimate bacterial viability counts.

Female C57BL/6J mice were used. All animals weighed 20 to 25 g and were provided by Harlan Ibérica, S. L. The animals were specific pathogen free and nourished *ad libitum* with sterile water and food. Before inoculation, the mice were anesthetized by intraperitoneal injection of ketamine and xylazine (100 mg and 10 mg per kg of body weight respectively). Forty microliters of the corresponding bacterial suspension, containing the indicated bacterial counts, were inoculated intranasally by pippeting directly in nasal orifices.

In addition to the evaluation of associated mortality and lung bacterial load, lung histopathology studies were performed. Briefly, lungs were fixed in 10% formalin, embedded in paraffin, sectioned (4 to 6 µm), and stained with hematoxylin and eosin. Lung inflammation was then blindly scored by an expert pathologist using the criteria of Johansen et al. [31] (1, normal; 2, mild focal inflammation; 3, moderate to severe focal inflammation with areas of normal tissue; 4, severe inflammation to necrosis and severe inflammation throughout the lung).

### Pulmonary infection survival model

Three groups of 10 mice each per strain were inoculated with two different bacterial inocula of 1×10^6^ and 5×10^5^ of bacteria per animal of strains PA14 and PA14*glpT::MAR2xT7* approximately, and mortality was recorded for 7 days. A Kaplan-Meier test was performed to determine the difference between mortality curves in the survival experiments. P values less than 0.05 were considered to be statistically significant.

### Estimation of spontaneous Fos-R mutation rates

For an estimation of the *in vitro* spontaneous mutation rate of PA14 and its derivative mutant *mutS::MAR2xT7* strains, approximately 10^3^ cells from overnight cultures were inoculated in 8 tubes, each containing 1 ml of LB, and subsequently incubated at 37°C with strong shaking for 16 h. Aliquots from successive dilutions or concentrated cultures were plated onto Mueller-Hinton agar (MHA) plates with fosfomycin (128 µg/ml). Additionally, appropriate dilutions with no antibiotic were plated onto MHA to estimate viability. The number of colonies growing after 24 h of incubation were determined. The mutation rate was then estimated using the Jones median estimator [32], and 95% confidence intervals were calculated using JONATOR (v0.1), a software developed in gfortran which implements an algorithm described elsewhere [33]. This software is free and available upon request. The same procedure was followed for an estimation of the mutation rate *in vivo*, but in this case, 8 mice were inoculated intranasally with approximately 5×10^5^ bacteria per animal from log-phase cultures of PA14 and the *mutS*-deficient strain. Lung homogenates were then obtained 48 hours postinfection, as described elsewhere. Proper dilutions of the lung homogenate were plated, as described for the procedure *in vitro*.

### Estimation of the frequencies of spontaneous mutants resistant to fosfomycin, tobramycin and imipenem

An estimation of the frequencies of spontaneous mutants resistant to fosfomycin (128 µg/ml), tobramycin (4 µg/ml), imipenem (4 µg/ml) and the combinations of tobramycin plus fosfomycin (4 and 128 µg/ml, respectively) and imipenem plus fosfomycin (4 and 128 µg/ml, respectively) was performed for the PA14 and *mutS::MAR2xT* strains, as previously described [24]. In the case of the *glpT::MAR2xT* strain, the mutant frequency was determined for tobramycin (4 µg/ml) and imipenem (4 µg/ml) to study the possible epistatic effects of resistance to fosfomycin and these two antibiotics.

### Evaluation of the fitness and *in vivo* selection of fosfomycin resistant mutants

Mice were inoculated with approximately 5×10^5^ cells of strains PA14, PA14*mutS::MAR2xT7* and PA14*glpT::MAR2xT7* in saline solution. Twenty-four hours after inoculation, two groups of 8 mice per strain were either treated with fosfomycin by receiving two intraperitoneal injections in saline solution with an interval of 8 hours (200 mg/kg/8 h) [34], [35], [36] or untreated. After 48 hours of inoculation (16 h after the end of the treatment in the case of fosfomycin-treated mice), the animals were sacrificed and their lungs aseptically extracted. The left lung was homogenized in 2 ml of saline using the Ultra-Turrax T-25 disperser (IKA, Staufen, Germany). Serial 1/10 dilutions were plated in MHA, and the total bacterial load was determined. To quantify Fos-R mutants, lung homogenates or serial 1/10 dilutions were placed in MHA plates containing Fos 128 µg/ml. The established lower limit of detection was 4 cfu of mutants per lung. The statistical differences among groups were estimated by a Kruskal-Wallis test for lung bacterial counts, and the Mann-Whitney U test was used to compare the proportion of Fos-R mutants between the group receiving fosfomycin and the untreated one. P values of less than 0.05 were considered to be statistically significant.

### Competition experiments

Sixteen mice were inoculated with a 1∶1 mix of PA14 and PA14*glpT::MAR2xT7* for a final inoculum size approximately of 5×10^5^. Twenty-four hours after inoculation, the mice were divided into two groups (8 mice each) and treated with Fos (200 mg/Kg/8 h) or untreated. After 48 hours of inoculation (16 h after the end of the treatment in the case of Fos-treated mice), the animals were sacrificed and their lungs aseptically extracted. The left lung was homogenized in 2 ml of saline using the Ultra-Turrax T-25 disperser (IKA, Staufen, Germany). Serial 1/10 dilutions were plated onto MHA and MHA with 10 µg/ml of gentamycin to determine the total bacterial load and the PA14*glpT::MAR2xT7* bacterial load, respectively. In a similar way, eight tubes were inoculated with approximately 10^3^ bacteria of each bacterial strain in 5 ml of LB and incubated at 37°C with agitation for 24 h. The bacterial counts were determined following the procedure described above for lung homogenate. The competition index (CI) was defined as the *glpT::MAR2xT7* mutant/wild-type ratio. The statistical analysis of the the CI values distribution was performed by a Mann-Whitney U test. P values of less than 0.05 were considered to be statistically significant.

### Analysis of Fos-R mutants generated *in vivo*

For the complementation studies 10 Fos-R mutants isolated from 10 different non-treated mouse lungs inoculated with the wild type strain, were complemented with the plasmid pBBR-*glpT*, as previously described [37]. The plasmid pBBR-*glpT* and its parental empty vector pBBR1MCS-3 [38] were introduced by electroporation into all the isolated Fos-R mutants. Transformants were selected on LB-agar supplemented with 150 µg/ml of tetracycline. The minimal inhibitory concentration (MIC) of fosfomycin was determined as described [39] for three colonies of every transformed mutant. The *glpT* gene of all mutants was sequenced, and compared with the wild type gene as described [37].

### Biofilms

An abiotic solid surface biofilm formation assay was performed in 96-well polystyrene microtiter plates in LB and minimal FBA [27] media in a humid chamber at 37°C, as described previously [40]. Biofilms were quantified after seven days of incubation. Forty independent replicas were carried out for each strain. Data were compared by a Student's t-test.

### Antibiotic susceptibility testing

Minimal inhibitory concentrations (MICs) of fosfomycin, tobramycin and imipenem were determined for the PA14 strain and its derivative mutants *mutS::MAR2xT7 and glpT::MAR2xT7* by the broth microdilution method, as recommended by the CLSI [39].

## Results

### *glpT* null mutant is as lethal as its parental strain

**Figure 1:**
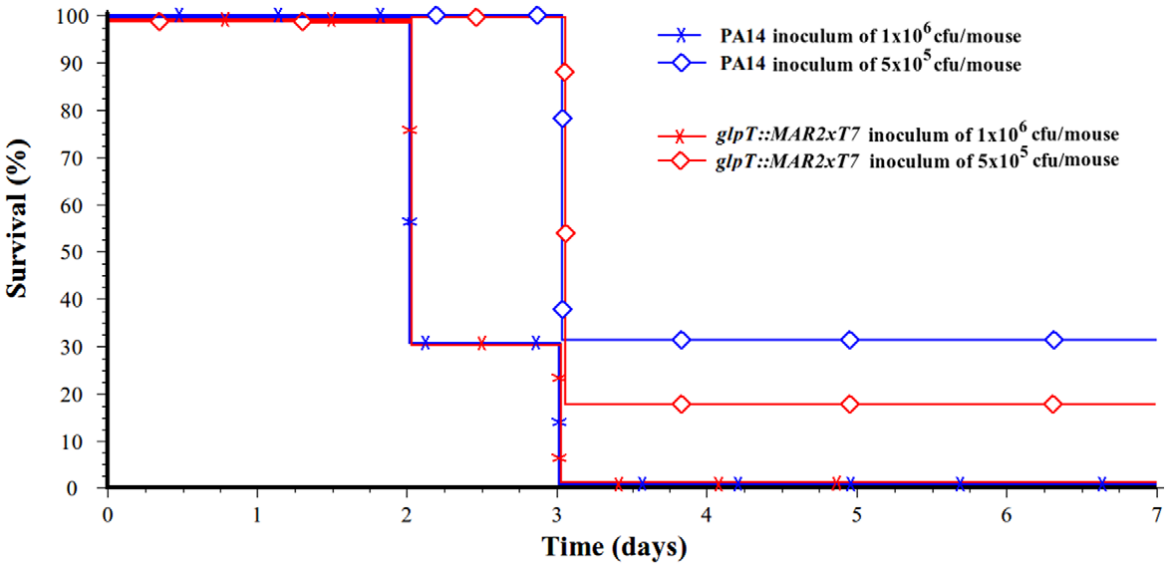
Mortality curves. Mice were infected with two different inocula of *P. aeruginosa* PA14 (blue lines) and its mutant *glpT::MAR2xT7* derivative (red lines) strains. The Kaplan-Meier analysis failed to detect any significant difference between curves from the same bacterial inoculum (1×10^6^ cell (crosses) and 5×10^5^ cells (diamonds)) (p = 0.81 and p = 0.21, respectively).

We did not detect any statistically significant difference in mortality between the PA14 wild type strain and the null mutant *glpT::MAR2xT7* (Fos-R), independently of the size of the inoculum (Figure 1). The Kaplan-Meier analysis revealed no discrepancy in the mortality curves between strains receiving the same inoculum (p = 0.81, p = 0.21, for approximately 1×10^6^ and 5×10^5^ bacteria, respectively). These results indicate that the disruption of the *glpT* gene does not interfere with the infective capacity and mortality caused by *P. aeruginosa* in this model.

### Effect of fosfomycin treatment in bacterial load of the wild type, Fos-R, and *mutS* strains

**Figure 2:**
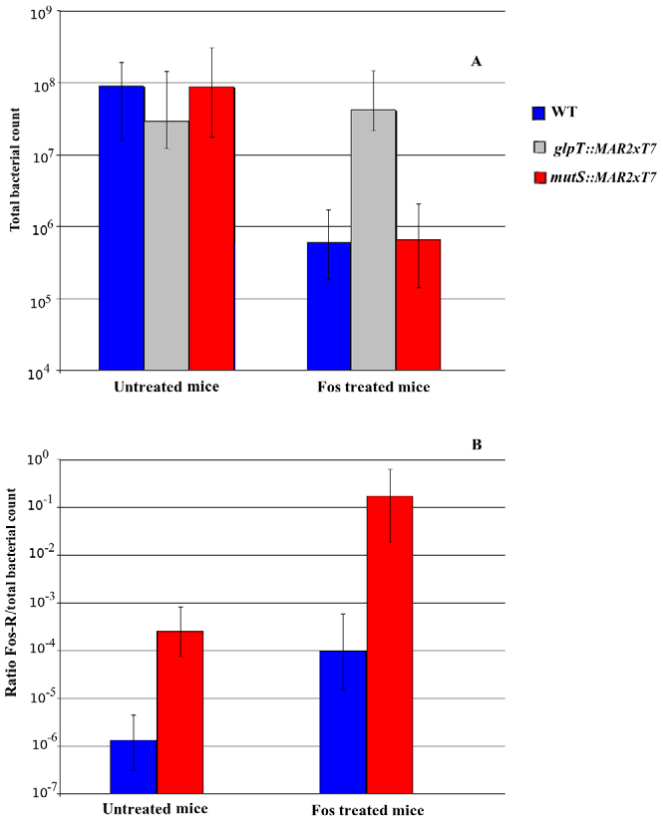
Recovery of bacteria from lungs 48 hours post infection. Mice were infected with approximately 5×10^5^ cfu/animal and treated with two doses of fosfomycin (200 mg/kg) 24 hours after inoculation or with no antibiotic. PA14 (blue columns) and its mutant derivatives *mutS::MAR2xT7* (red columns) and *glpT::MAR2xT7* (gray columns) (A). Ratio of Fos-R mutants/total bacteria in lungs from treated and non-treated mice (B). Values are medians and error bars represent interquartile ranges.

The colonization of mice lungs by *P. aeruginosa* PA14 (median 9.16×10^7^, interquartile range from 1.57×10^7^ to 1.73×10^8^) forty eight hours post infection did not show statistical differences in the number of colony-forming units (cfu) per mouse, in comparison with the other two strains, *mutS::MAR2xT7* (median 8.86×10^7^, interquartile range from 2.15×10^7^ to 2.55×10^8^) and *glpT::MAR2xT* (median 2.95×10^7^, interquartile range from 1.12×10^7^ to 1.30×10^8^) (p = 0.44). On the other hand, when mice were treated with fosfomycin, bacterial counts of the wild type strain (median 6.02×10^5^, interquartile range from 2.65×10^5^ to 1.66×10^6^) and the *mutS*-deficient mutant (median 6.60×10^5^, interquartile range from 1.18×10^5^ to 2.06×10^6^) were dramatically decreased by two orders of magnitude (p = 0.005 and p = 0.009, respectively). However, the *glpT::MAR2xT* mutant did not suffer any drop in bacterial counts (median 4.19×10^7^, interquartile range from 2.01×10^7^ to 1.06×10^8^) with no differences with the non-treated experiment (p = 0.91) (Figure 2A).

The proportion of resistant mutants is greatly increased in the wild type strain after fosfomycin treatment (Figure 2B), especially if we consider that this increase appeared only after 24 hours from the time the antibiotic was administered. The untreated group has the expected proportion of Fos-R mutants according to its mutation frequency (median 1.58×10^−6^, interquartile range from 3.62×10^−7^ to 5.69×10^−6^). However, the proportion of wild type Fos-R cells increased by two orders of magnitude (median 9.82×10^−5^, interquartile range from 1.56×10^−5^ to 5.76×10^−4^), which represents a more than 60 fold increase and is statistically significant (p = 0.009) (Table 1). Furthermore, for the PA14 *mutS::MAR2xT7* strain this phenomenon was agravated. While the proportion of resistant mutants in untreated mice was as expected (median 2.54×10^−4^, interquartile range from 8.27×10^−5^ to 7.71×10^−4^), in treated mice the proportion increased up to two fosfomycin resistant mutants for every ten bacteria (median 1.70×10^−1^, interquartile range from 2.71×10^−2^ to 7.15×10^−1^), which corresponds to a greater than 600 fold increase and is statistically significant (p = 0.001) (Table 1).

**Table 1:** Amplification of fosfomycin resistant mutants in mice treated with the antibiotic.

Strains	FosR mutant/total cfu (treated mice)	FosR mutant/total cfu (untreated mice)	Fold increase in Fos-R
PA14 WT	9.8×10^5^	1.58×10^6^	61.98
*mutS::MAR2xT7*	1.70×10^1^	2.54×10^4^	666.76

Fold increase values were calculated as the ratio between the median of the proportion of Fos-R mutants/total bacterial counts (figure 2B) of treated and untreated mice.

One interesting point is that the median of the absolute number of mutants actually remains constant if we compare the Fos-R mutant load in mice receiving fosfomycin or not, although the total bacterial count decreased by two orders of magnitude in the dosed mice. This indicates that from the start of fosfomycin dosing, probably because the bacterial load was very high at that point, there is a multiplicity problem that limits the amount of mutants. However, when mice are dosed, the number of fosfomycin-susceptible bacteria diminish, thus the resistant ones can prevail at a higher rate in comparison with the non-treated group.

### Mutation rates to fosfomycin resistance *in vivo* are similar to those *in vitro*

**Figure 3:**
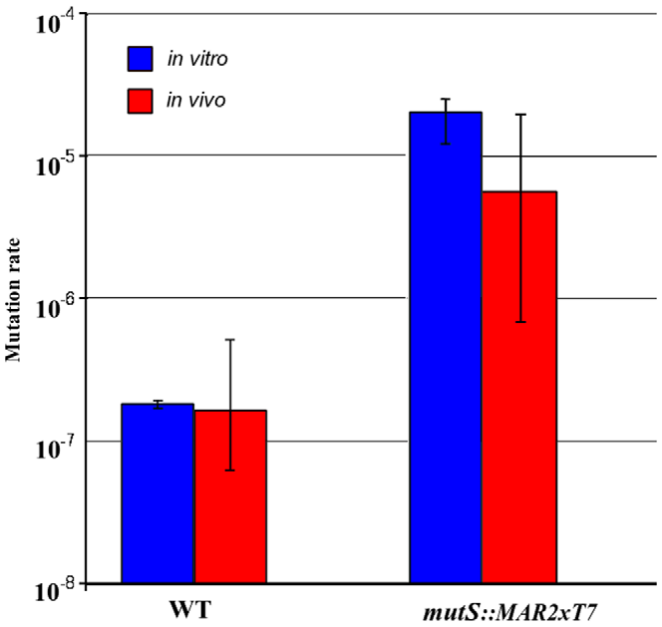
Mutation rates. *in vivo* (acute mice lung infection model, red columns) and *in vitro* (blue columns) of *P. aeruginosa* PA14 and its mutant derivative *mutS::MAR2xT7* strains are shown. Bars represent confidence intervals at a signification level of 0.05.

Results shown in figure 3 indicate that the mutation rates obtained *in vitro* and *in vivo* are very similar (95% confidence interval). As expected, mutation rates to fosfomycin resistance are in agreement with the previously described values for these *P. aeruginosa* strains [24]. The values were around 10^−5^ for the *mutS*-deficient mutant, while the parental strain showed values two orders of magnitude lower for both the *in vitro* and the *in vivo* experiments. The high dispersion in the data corresponding to the *in vivo* experiment is notable and probably due to the innate variation of the animal models compared to the controlled laboratory conditions of the *in vitro* experiments. These results indicate that there is no limitation in the emergence of *in vivo* Fos-R mutants in *P. aeruginosa*.

### Wild type strain did not outcompete the Fos-R mutant *in vivo* or *in vitro*

The *in vitro* competition experiment rendered no statistical differences between the WT strain and *glpT* mutant (CI = 1.04; p = 0.80). Similarly, in the *in vivo* competition experiment in mice which did not receive fosfomycin treatment, the competition index between the wild type strain and *glpT*-deficient mutant, was 0.78, indicating no fitness advantage in any strain (p = 0.22). As expected, the scenario changed totally when the mice were treated with fosfomycin. In this case the CI rose to 9.70 in favour of the Fos-R mutant (p = 0.02), showing that Fos-R mutants will prevail in the population when pressure is exerted by fosfomycin.

### All Fos-R mutants generated *in vivo* contain mutations in the *glpT* gene

Complementation studies showed that *glpT* mutations were the cause of fosfomycin resistance *in vivo* in all cases. Ten randomly chosen mutants from the wild type strain recovered fosfomycin sensitivity upon complementation with the *glpT* wild type gene. These results coincide with those from our previous work [37]. The sequence analysis revealed different mutations in the *glpT* gene (Table 2). The mutations consisted mainly of single-base deletions (5 mutants), which caused inactivation of the gene by frameshifts. There were also deletions of 2, 6 and 12 pb causing frameshifts or loss of 3 or 4 aminoacids respectively. Additionally, a G to A transition was detected at position 900 of the ORF.

**Table 2:** Mutations in *glpT* of ten randomly chosen spontaneous mutants of *P. aeruginosa* PA14 selected from *in vitro* and *in vivo* experiments.

Inactivating mutations of *glpT in vitro* and *in vivo*			
*in vitro* [37]		*in vivo* (this work)	
Mutant No.	Mutation position	Mutant No.	Mutation position
FosR-7	A_59_ deletion, 1 bp (frameshift)	FosR-5	T_215_CGCCA_220_ deletion 6 bp
FosR-9	C_219_ATCGC_225_ deletion, 6 bp	FosR-6	T_221_CGCCT_227_ deletion 6 bp
FosR-1	A_220_TCGCC_226_ deletion 6 bp	FosR-4	T_226_A_227_ deletion, 2 bp (frameshift)
FosR-2	T_365C_ATGTT_371_ deletion, 7 bp (frameshift)	FosR-9	T_377_ deletion, 1 bp (frameshift)
FosR-3	G_410_ to A transition	FosR-10	G_619_ deletion, 1 bp (frameshift)
FosR-5	G_596_ deletion, 1 bp (frameshift)	FosR-3	A_869_ deletion, 1 bp (frameshift)
FosR-10	C_975_GG insertion, 3 bp	FosR-2	T_887_ deletion, 1 bp (frameshift)
FosR-4	A_1006_ to C transition	FosR-7	G_900_ to A transition
FosR-8	C_1086_ to G transition	FosR-8	C_1032_GGCAACCCGGC_1043_ deletion 12 bp
FosR-6	G_1098_ to A transition	FosR-10	T_1231_ deletion, 1 bp (frameshift)

Mutants were arranged according to the nucleotide position using the A of the ATG of the ORF as reference.

### Inactivation of *glpT* does not affect biofilm formation *in vitro*

**Figure 4:**
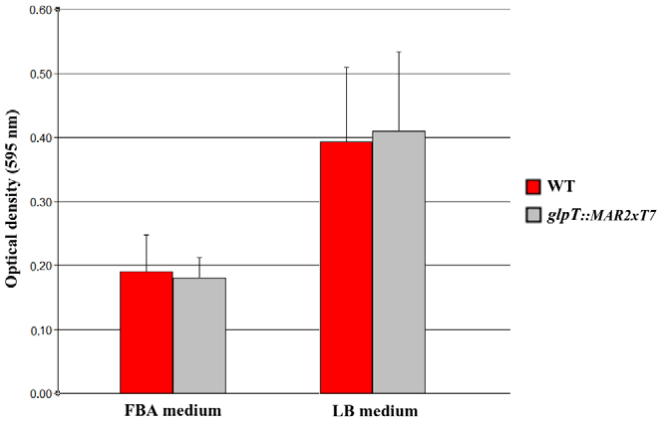
Seven-day biofilm formation. Biofilm formation of *P. aeruginosa* PA14 (red columns) and its mutant derivative *glpT::MAR2xT7* (gray columns) is shown. The values are represented as the mean ± SD. Forty replicas were performed per strain and medium. No statistically significant differences were detected between strains for each medium (FBA, p = 0.31; LB, p = 0.59).

We could not detect any statistically significant difference between the wild type strain and its *glpT::MAR2xT* derivative in biofilm formation. Figure 4 shows the ability of the two strains to form biofilm in FBA (mean ± standard deviation: 0.19±0.07 for the wild type and 0.17±0.05 for the *glpT*-deficient mutant, p = 0.31) or LB (mean ± standard deviation: 0.39±0.12 for the wild type and 0.41±0.13 for the *glpT*-deficient mutant, p = 0.59).

### Histopathology studies

**Figure 5:**
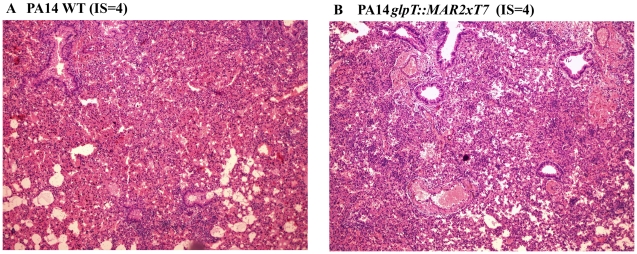
Histology of murine lungs after infection with *P. aeruginosa*. Shown are representative examples of the histology results obtained for murine lungs 48 hours after infection with the *P. aeruginosa* strain PA14 (A) or PA14*glpT::MAR2xT7* (B). IS, inflammatory score determined as described by Johansen et al. [31].

Inflammation was scored in 10 mice per strain by using the criteria of Johansen et al. [31]. In summary, both strains (PA14 and PA14*glpT::MAR2xT7*) induced a very high level of inflammation (3.9±0.32 and 3.6±0.97; mean and standard deviation of scores for PA14 and PA14*glpT::MAR2xT7*, respectively) with no significant differences between them (p = 0.40). Most of the infected mice (90% and 80% for PA14 and PA14*glpT::MAR2xT7*, respectively) had a score of 4 (from severe inflammation to necrosis and severe inflammation throughout the lung) (Figure 5).

### Frequency of emergence of resistant mutants to combined antibiotics

To investigate the possibility of appearance of double mutants with resistance to the combinations of fosfomycin with different antibiotics, we first determined the MICs of these antibiotics for the PA14 strain and its derivatives *mutS::MAR2xT7* and *glpT::MAR2xT7* (Table 3). The mutant frequencies of resistance to the combinations of tobramycin plus fosfomycin (4 and 128 µg/ml, respectively) and imipenem plus fosfomycin (4 and 128 µg/ml, respectively) were studied. Obviously, the frequencies of mutants resistant to the single antibiotics fosfomycin (128 µg/ml), tobramycin (4 µg/ml) and imipenem (4 µg/ml) were also calculated. Table 4 shows that the frequency of mutants resistant to the combination of fosfomycin with tobramycin is below our limit of detection (≤10^−11^), making improbable the emergence of resistance to both antibiotics when administered together, even in a hypermutable background. However, the frequency of mutants resistant to the combination of imipenem plus fosfomycin is relatively high in the wild type (2.1×10^−9^) with a 2-log increase in the hypermutator mutant (1.1×10^−7^). These results suggest that the generation of mutants resistant to the combination of fosfomycin with some antibiotics may not be very difficult for *P. aeruginosa*. Interestingly, the frequencies of mutants resistant to either tobramycin or imipenem of the *glpT*-deficient strain were similar to those of the wild type. Thus, there is apparently no epistatic effect between the resistance determinants of the pairs tobramycin/fosfomycin and imipenem/fosfomycin.

**Table 3:** Minimal inhibitory concentrations of the antibiotics fosfomycin, tobramycin and imipenem for PA14 and its derivative mutants *mutS::MAR2xT7 and glpT::MAR2xT7*.

Antibiotic	MIC (µg/ml)		
	wt	*mutS::MAR2xT7*	*glpT::MAR2xT7*
Fosfomycin	8	8	1024
Tobramycin	0.25	0.25	0.25
Imipenem	0.5	0.5	0.5

**Table 4:** Frequency of mutants resistant to single antibiotics and the combinations of fosfomycin with tobramycin and imipenem.

Antibiotic	Concentration a	Mutant frequency	
		wt	*mutS::MAR2xT7*
Tobramycin	4	2.2×10^−9^	1.4×10^−7^
Imipenem	4	2.3×10^−6^	9.1×10^−4^
Fosfomycin	128	1.5×10^−6^	1.1×10^−4^
**Combination**			
Tobramycin +Fosfomycin	4+128	<10^−11^	<10^−11^
Imipenem +Fosfomycin	4+128	2.1×10^−9^	1.1×10^−7^

a: antibiotic concentation in µg/ml.

## Discussion

Among the different factors contributing to the ascent of antibiotic resistance, fitness cost is now recognized as prominent and one that ought to be evaluated when assessing the suitability of any drug treatment [8], [10]. Here we have shown that in the absence of fosfomycin, the *P. aeruginosa* PA14 wild type strain (sensitive) was unable to outcompete the null *glpT* mutant (Fos-R strain) after two days of *in vivo* or 24 hours of *in vitro* competition. Similar results were obtained previously in an *in vitro* experiment comparing growth rate in minimal media [37].

On the other hand, after two days of acute lung infection, the bacterial lung counts and the ability of the *glpT* mutant to cause death did not differ from the parental strain. These results clearly indicate that fosfomycin resistance does not exert a significant burden on fitness or virulence for *P. aeruginosa*.

This divergence from the *E. coli* patterns could be explained to some extent by the fact that, although both species share the reduction in uptake of fosfomycin on the basis of resistance mechanism [8], [14], [15], [16], [37], they apparently differ in the specific target genes for mutation and their metabolic implications. It is well established that, in *E. coli* and other gram negative species fosfomycin access primarily through the L-alpha-glycerol-3-phosphate transport system (*glpT*), and to a lesser extent via the hexose phosphate transport system (*uhpT*) [15]. Moreover, these two genes are known to be positively regulated by cAMP, and so mutations that lower the cAMP level in the cell (i.e. in the *cyaA* and *ptsI* genes) can induce resistance as well [41]. This reduction in cAMP levels can produce profound alterations to the bacterial metabolism, and can probably explain the significant reduction in fitness and virulence shown for many resistant isolates in *E. coli* [8]. Aldoght, Fos-R clinical isolates of *E. coli* are now being found and suggesting that this is a viable threat [9], [21].

In contrast, the uptake of fosfomycin in *P. aeruginosa* depends exclusively on GlpT permease [37], which does not seem to be regulated by cAMP levels. Indeed, the *glpT* gene is isolated in the *P. aeruginosa* genome (www.pseudomonas.com), while the *E. coli* ortholog is included inside the well-known *glg* operon [42]. This may be related to another radical difference between these two species, as has been stated in the case of *P. aeruginosa* which displays a higher preference for glycerol as carbon source than for glycerol-3-phosphate [43]. These observations could be understood considering the different ecology of both species.

We have also performed an analysis of the *glpT* sequences from Fos-R mutants obtained *in vivo* and complementation studies, which show that fosfomycin resistance is acquired in *P. aeruginosa* by any mutation capable of inactivating GlpT activity. From a comparative point of view with *in vitro* generated mutants, mutational pathways appear to be very similar in both conditions.

Our results have at least two relevant implications from a medical stand point. First, our data demonstrate that the fitness cost of antibiotic resistance needs to be measured for each particular species of interest, and that the observations made in just one species cannot be extrapolated to another, since the genetic particularities might modulate the effects of bacterial fitness. Moreover, fosfomycin resistant clinical isolates of *E. coli* are now being found, suggesting that this is a viable threat [9], [21]. Second, our results curb the good prospects that fosfomycin has recently promised, at least for its use on *P. aeruginosa* infections. Similarly, it has been reported that there is no repercussion for fitness in any other resistance mechanism mediated by gene inactivation in *P. aeruginosa*. A partial deregulation of *ampC* expression by mutation of its repressor *ampD* confers efficient resistance to cephalosporins, without biological cost, due to two other copies of *ampD* [44]. However, contrarily to what occurs with *glpT* mutations, where a very high level of resistance is conferred, the *ampD*-based resistance is far from being complete.

The high proportion of fosfomycin mutants generated by the hypermutable *mutS*-deficient strain indicates a real danger of rapid emergence of resistant mutants during a treatment. This is especially true in chronic respiratory infections, where one of the most prevalent and dangerous bacterial pathogens is *P. aeruginosa* [45]. In these infections the prevalence of hypermutable strains is abnormally high [46] and have been linked to antibiotic resistance development and persistence in respiratory airways [47], [48]. Thus, one can expect that in such infections fosfomycin resistance would develop as soon as patients begin the treatment.

As fosfomycin has been proposed as potentially useful in combined therapy [49], we studied *in vitro* the probability of acquisition of resistance to the combination of fosfomycin with two different antibiotics, tobramycin and imipenem. While the combination of fosfomycin with tobramycin makes improbable the emergence of resistance to both antibiotics when administered together, even in an hypermutable background, the results from the combination of fosfomycin plus imipenem suggest that the generation of mutants resistant to both antibiotics is not very difficult for *P. aeruginosa*. However, additional studies are still needed to determine the real effectiveness of fosfomycin when administered together with these and other antibiotics. Recently Ward et al [50] have reported that the biological cost of resistance to a second antibiotic in *P. aeruginosa* could be greater than that of previously acquired resistance. Finally, the total cost depends on the genetic background in which resistance evolve and its interaction with the environment. Well conducted studies are needed to find a good combination of antibiotics where double resistance, if available, has a sufficiently high cost to impede the persistence of infection.

We were unable to identify any condition where the inactivation of *glpT* has some significant repercussion on *P. aeruginosa* fitness. Biofilm formation of the defective mutant did not show any alteration when compared with the wild type strain, which probably indicates that this gene does not influence biofilm development. These results are consistent with the fact that some metabolic genes are down regulated during biofilm formation [51], [52].

In conclusion, apart from the absence of *in vivo* fitness cost of fosfomycin resistance in *P. aeruginosa*, our findings reveal that we need a deeper understanding of the interplay between antibiotic resistance, biological fitness and virulence. This knowledge will be invaluable if we aspire to improve the development of drug antibiotherapy for the different pathogens nowadays threatening humankind.
